# Identification of cost-effective biosecurity measures to reduce *Salmonella* along the pork production chain

**DOI:** 10.3389/fvets.2024.1380029

**Published:** 2024-03-18

**Authors:** Clara Bester, Annemarie Käsbohrer, Neil Wilkins, Guido Correia Carreira, Tatiana Marschik

**Affiliations:** ^1^Centre for Food Science and Veterinary Public Health, Clinical Department for Farm Animals and Food System Science, University of Veterinary Medicine, Vienna, Austria; ^2^Unit Epidemiology, Zoonoses and Antimicrobial Resistance, Department Biological Safety, German Federal Institute for Risk Assessment, Berlin, Germany; ^3^Department of Epidemiological Sciences, Animal and Plant Health Agency, Addlestone, United Kingdom

**Keywords:** biosecurity measures, cost-effectiveness, food safety, pork production chain, *Salmonella*

## Abstract

The continued occurrence of salmonellosis cases in Europe attributed to the consumption of pork products highlights the importance of identifying cost-effective interventions. Certain biosecurity measures (BSMs) may be effective in reducing the prevalence of specific pathogens along the pork production chain and their presence in food products. The objective of this study was to identify pathogen-specific, cost-effective BSMs to reduce Salmonella at different stages of the pork production chain in two European countries - Austria (AT) and the United Kingdom (UK). For this purpose, a cost-benefit analysis was conducted based on the epidemiological output of an established quantitative microbiological risk assessment that simulated the implementation effect of the BSMs based on their risk ratios. For each of the BSMs, the associated costs and benefits were assessed individually and country-specifically. For both AT and UK, nine different BSMs were evaluated assuming a countrywide implementation rate of 100%. The results showed that four BSMs were cost-effective (benefit-cost ratio > 1) for AT and five for the UK. The uncertainty regarding the cost-effectiveness of the BSMs resulted from the variability of individual risk ratios, and the variability of benefits associated with the implementation of the BSMs. The low number of cost-effective BSMs highlights the need for holistic risk-based models and economic assessments. To increase the willingness to implement BSMs and maximize the benefits for stakeholders, who carry the majority of the implementation costs, epidemiological assessments of BSM effectiveness should consider the impact on several relevant pathogens simultaneously.

## 1 Introduction

The European Union’s (EU) food safety policy aims to ensure high standards of consumers’ health protection (1, 2). Despite increased efforts to reduce pathogens in the food production chain, foodborne outbreaks and illnesses continue to occur, and food safety has been increasingly recognized as one of the main aspects of public health (3–5). Pigs represent the largest livestock category reared across the EU and pork accounts for nearly half of the total meat produced in the Common Market (6). Pigs are an important reservoir of certain *Salmonella enterica* spp. serovars (SAL) which are known causes of foodborne diseases in humans (7). While Commission Regulation (EC) 2073/2015 defines the relevant food safety procedures, including process-hygiene criteria, and general instructions for surveillance of SAL are given through Council Regulation (EC) 2,160/2003, specific regulations demanding mandatory programs for SAL in pigs do not exist across the EU (8). Nevertheless, illnesses in humans caused by SAL in Europe are associated with substantial economic losses resulting in up to €90 million annually (9). Additionally, SAL has been one of the most commonly diagnosed foodborne pathogens for decades (7). While the total number of human salmonellosis cases exceeded 60,000 in 2020, only infections with specific serovars, such as *S. typhimurium* or *S*. Derby, have been attributed to the consumption of pork products (7). However, the pig reservoir has been reported as the second most important source of human salmonellosis after laying hens in the EU (10, 11). Salmonellosis in humans, which is usually characterized by gastroenteritis is mostly foodborne (12), but people having direct contact with pigs are also at increased risk of contracting the disease (13).

SAL contamination may occur at different points of the production cycle, either at the primary production level or in further processing, including slaughtering (10). Biosecurity standards and hygiene management in pig farms play a very important role in preventing the introduction of many pathogens as well as reducing their spread within the farm once the agent has been introduced (14, 15). Broadly, biosecurity relates to the implementation of measures that can reduce the transmission, introduction, establishment or survival of a pathogen (16). External and internal BSMs target either aspects of farm management, such as replacements, breeding strategies, and wildlife near farms, or focus on herd practices, carcass disposal, cleaning protocols and personnel hygiene (15, 17). When deciding on employment of specific BSMs, farm characteristics, such as location, facilities, production type and herd size need to be taken into account in addition to country-specific production and management strategies (14, 17, 18). Nevertheless, biosecurity plans should be designed for individual farms considering that the BSM’s effectiveness is usually recommended in general terms (14, 19), and evaluated in field studies based on identifying correlations with decreased (sero-) prevalence. While agreement exists for the positive impact of some BSMs, such as a low number of potential sources for SAL, e.g., for the purchase of new livestock (14), or quarantine protocols for breeding sows (17), the overall effectiveness of many BSMs has not yet been described in the scientific literature. In addition, there is a lack of standardized on-farm BSM implementation protocols (17). Considering the shift in pig production from smaller to large holdings, reliable disease prevention has become increasingly more relevant (15). Nevertheless, the employment of sustainable biosecurity strategies and their continuous improvement remain challenging tasks for many pig farms (20).

The effectiveness of BSMs to mitigate specific pathogens is poorly understood (21). Epidemiological models are recognized as valuable tools that can assist decision-makers in identifying and evaluating strategies for disease control (13, 22, 23). Only a few simulation models have been used to investigate the effect of specific BSMs on the occurrence of infectious diseases in the pork production chain (PPC) (24–27). To assist EU Member States, a quantitative microbiological risk assessment (QMRA) model for SAL was developed and established (13). Dependent on country-specific production systems and infection prevalence in herds, it allows assessment of the effectiveness of on-farm and slaughterhouse interventions in reducing SAL in pigs and humans. In addition, it explores the epidemiological processes in the PPC and offers valuable insight into a pathogen’s transmission, including exposure to humans (13, 25, 28).

The implementation of BSMs at various specific points of the PPC is associated with considerable costs due to, e.g., required manpower, equipment, material, installation, and maintenance. Identifying and quantifying these costs are important to justify and prioritize investments and to inform stakeholders (29). The benefits associated with biosecurity efforts are reflected in their potential to reduce the pathogen’s prevalence and thereby prevent losses caused by animal and human diseases. The various aspects of the associated human diseases are often captured by the Cost of Illness (COI) methodology. It encompasses monetary factors, including direct, e.g., diagnostic or treatment, and indirect expenditures that result from, e.g., premature death or disability to work (30, 31). Different economic methods are available to determine the cost-effectiveness of BSM implementation. The cost-benefit analysis (CBA), which has been widely used in economic assessments of animal disease control programs and policies, represents a validated approach providing information on economic efficiency (32–34). It weighs the total benefit expressed in monetary terms against the total cost spent on BSM implementation and evaluates, through the benefit-cost ratio (BCR), the cost-effectiveness of a respective BSM (24, 35–37).

This study was conducted within the One Health EJP BIOPIGEE Group, which focuses on the improvement of biosecurity practices in pig farming across Europe. The BSMs evaluated in this study were identified through a literature review and meta-analysis carried out in the recently finalized corresponding sub-project (38). The objective of this study was to conduct a comprehensive economic assessment of the costs and benefits associated with those BSMs proven as useful in reducing SAL prevalence and to evaluate their cost-effectiveness along the PPC. This has been done for two European countries with different levels of SAL prevalence and livestock characteristics, i.e., Austria (AT) and the United Kingdom (UK).

## 2 Materials and methods

### 2.1 QMRA-based evaluation of the effectiveness of BSMs

**Figure 1:**
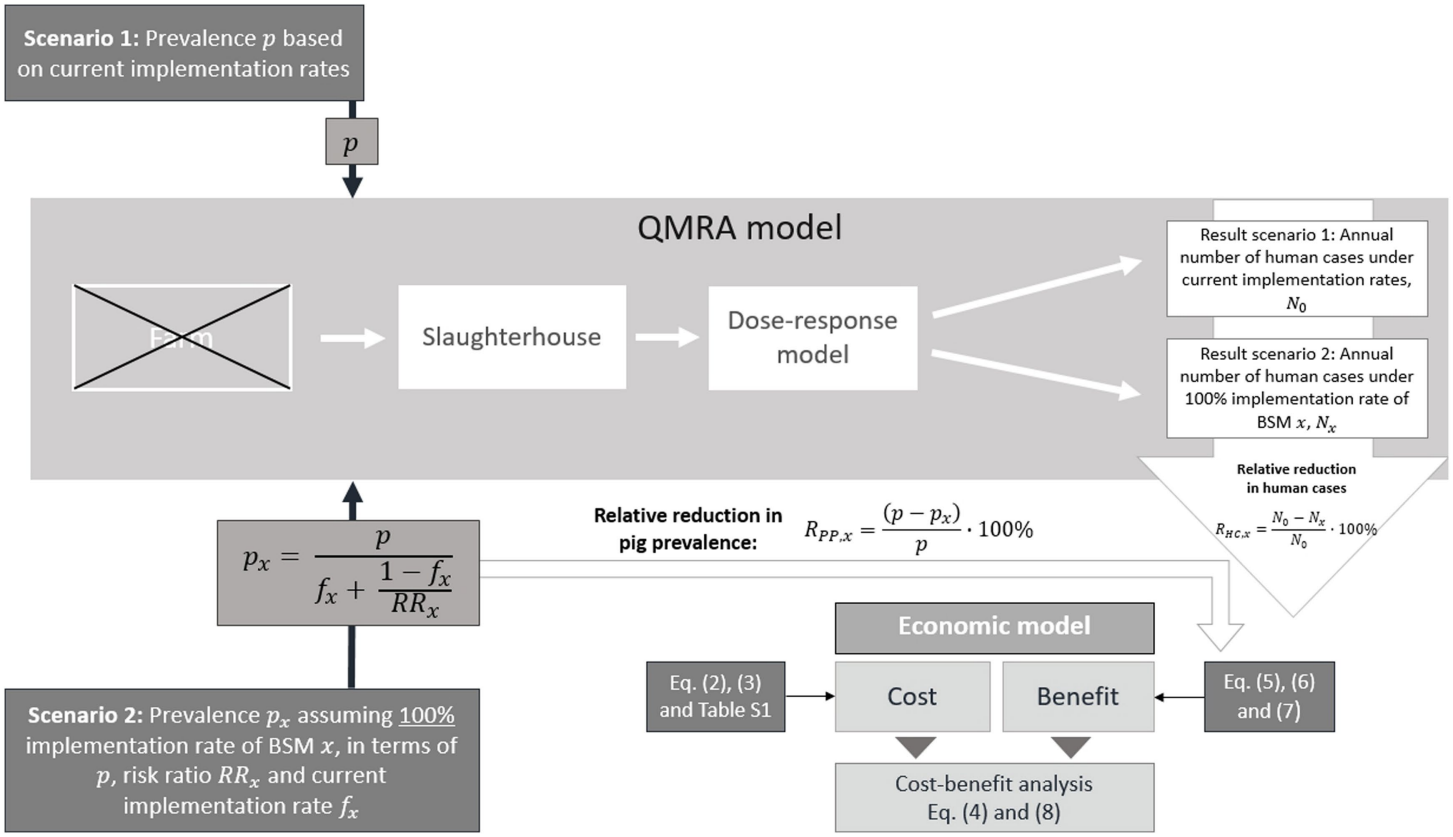
Concept of the cost-benefit analysis based on Quantitative Microbiological Risk Assessment (QMRA) model outputs for *Salmonella* (SAL)-specific effective biosecurity measures (BSMs). The cost-benefit analysis builds on a comparison of the output from the QMRA model, i.e., the annual number of human salmonellosis cases, for two different scenarios. Scenario 1 considers the current implementation rate for a given BSM x and the corresponding current slaughter pig prevalence p . Scenario 2 considers the 100% countrywide implementation rate for a given BSM x , which translates into a reduced prevalence in the finisher pig population $p_{x}$ . The reduction of the prevalence takes into account the BSM risk ratio $RR_{x}$ , and its current implementation rate $f_{x}$ . The QMRA model generally includes a farm module, which was not considered in this study. The output of the farm module (SAL prevalence among farm pigs) was replaced by the values p , and $p_{x}$ for scenario 1 and 2, respectively.

The concept of the study presented here was based on several steps (Figure 1), which are described in detail in the following sections. The literature-based selection of the BSMs targeting SAL was determined by the identified odds ratios (ORs) suggesting high pathogen-reduction-effect (i.e., upper limit of identified OR’s 95% confidence interval (CI) < 1), and the possibility to capture the implementation of the respective BSM in monetary terms (38). Thus, nine BSMs were selected for further economic evaluation (Table 1). To meet the methodological requirements of the QMRA model, the identified ORs were converted into risk ratios (RRs) (Supplementary Information 1.1).

**Table 1:** Biosecurity measures (BSMs) identified as effective in the reduction of *Salmonella* prevalence along the pork production chain.

BSM-specific data				AT (Baseline pig prevalence: p = 2%)		UK (Baseline pig prevalence: p = 32%)	
BSM	Description	RR^a^ median [95% CI]	Implementation rate^b^ $f_{x}$ (%)	Pig prevalence reduction^c^ $R_{\mathrm{pp},x}$ (%)	Human incidence reduction $R_{HC,x}$ (%) median [95% CI]	Pig prevalence reduction^c^ $R_{\mathrm{pp},x}$ (%)	Human incidence reduction $R_{HC,x}$ (%) median [95% CI]
Organic acid in feed, fattener	Addition of organic acid to pigs’ feed during the entire fattening period	0.783 [0.724;0.848]	38	15	18 [13;22]	14	15 [13;16]
Organic acid in feed, weaner	Addition of organic acid to pigs’ feed during the entire weaning period	0.528 [0.300;0.931]	43	33	35 [31;39]	33	32 [31;33]
Organic acid in water	Addition of organic acid to pigs’ water during the entire weaning period	0.250 [0.088;0.710]	5	73	73 [71;76]	74	73 [72;73]
Anal plugging	Application of an anal plug for each individual pig before scalding	[not applicable^d^]	0	n/a	93 [92;95]	n/a	93 [92;93]
Boot disinfection	Provision of boot disinfection trays between each section	0.799 [0.713;0.896]	27	15	14 [10;18]	15	14 [13;16]
Disinfection of farrowing pens	Disinfection of farrowing pens after each farrow	0.465 [0.302;0.716]	60	32	33 [29;37]	31	30 [29;31]
Rodent control	Rodent control performed by a professional company every 4–6 weeks	0.541 [0.372;0.788]	49	30	29 [24;33]	30	30 [29;31]
Vaccination^e^	Vaccination of sows and piglets	0.363 [−]	0	64	63 [60;67]	64	62 [62;63]
Vehicle wheel disinfection	Disinfection of vehicle wheels upon arrival at the farm	0.452 [0.293;0.699]	0.05 (AT) 73 (UK)	55	54 [51;58]	24	22 [21;24]

The corresponding risk ratio (RR), and the country-independent (unless stated otherwise) implementation rate were used to calculate the relative reduction in pig prevalence. Based on this reduced prevalence and further country-specific data, the impact on the reduction in the number of human cases was simulated in the Quantitative Microbiological Risk Assessment (QMRA) model and used as a basis for the cost-effectiveness assessment of the respective BSMs in Austria (AT) and the United Kingdom (UK). CI, confidence interval.

a Data for risk ratio calculation (38, 39) and implementation rate estimation (40) were provided from related BIOPIGEE sub-projects.

b Data for risk ratio calculation (38, 39) and implementation rate estimation (40) were provided from related BIOPIGEE sub-projects.

c The reduction in the pig prevalence is calculated based on a mathematical equation (Supplementary Information 1.2) and thereby presented without a range.

d No RR was used in this case. The BSM effect was simulated directly by QMRA, by setting the fecal leakage (anus) rate along the slaughter line to zero.

e Effect of vaccination against Salmonella in breeding sows and offspring according to Peeters et al. (41).

A SAL-specific farm-to-fork QMRA model described comprehensively elsewhere (13, 28), was used to simulate the impact of BSM implementation on the incidence of human cases in AT and the UK. To achieve this goal, the QMRA was run for two scenarios: Scenario 1 assumed the current BSM implementation rate which had been estimated based on a questionnaire analysis conducted as part of a related BIOPIGEE sub-project (40) (Table 1); Scenario 2 assumed a 100% countrywide on-farm implementation rate. The comparison of the QMRA model outputs, i.e., the number of salmonellosis cases in humans for both scenarios, allowed to estimate the impact of a countrywide implementation of the individual BSMs on human incidence (Figure 1).

The QMRA is divided into a series of modules (farm, slaughterhouse, dose-response), with the output from each module as the input to the next. The parameterisation of the QMRA model followed that of its previous versions (24, 25), whereas the farm module was excluded. Its usual output, i.e., the prevalence of SAL infected slaughter pigs, was replaced by prevalence values determined separately outside the model for both scenarios. For Scenario 1, the current slaughter pig prevalence (AT 2%; UK 32%) was estimated from the literature (40, 42). For Scenario 2, where a 100% countrywide on-farm implementation rate was assumed, this prevalence was reduced based on the RR, and the existing BSM implementation rate for each individual BSM (Supplementary Information 1.2). In short, under consideration of the 100% countrywide implementation rate of a BSM x, the effectiveness-determined new prevalence $p_{x}$ was estimated using the following equation (Eq. 1):


(1) $$p_{x}=\frac{p}{f_{x}+\frac{1-f_{x}}{RR_{x}}}$$

where p is the known slaughter prevalence, i.e., 2% in AT and 32% in the UK (42, 43), $f_{x}$ is the proportion of farms currently implementing a specific BSM x, and $RR_{x}$ is the identified risk ratio (Table 1). The remaining parameters for subsequent QMRA modules, e.g., relating to heat treatment, viral loads and dose-response, were sourced from the literature (28).

Except for the excluded farm module, all consecutive modules of the QMRA, i.e., transport and lairage (10,000 iterations), slaughter and processing (10,000 iterations), and consumption (10,000 iterations) were run for both scenarios and for each BSM. The number of iterations run was enough to ensure sufficient convergence (13). The simulation outputs of both scenarios delivered estimates on the number of annual human cases based on the probability of infection from consuming three different pork products, which were pork cuts, minced meat, and fermented ready-to-eat sausages. The probability of infection referred to the risk of infection at ingestion of one individual dose, which had been extrapolated considering national consumption patterns, such as *per capita* consumption of pork and proportion of sausages consumed, and population data (13, 28). The incidence values of both scenarios were compared to express the relative reduction in human incidence used to calculate the benefits resulting of a 100% BSM implementation rate (Figure 1).

Note that for anal plugging a different approach was used to determine its effect on human salmonellosis cases. The effect of anal plugging was simulated in the slaughterhouse module of the QMRA by changing the model parameter which describes the leakage of feces through the anus. In other words, the impact of anal plugging was not considered at the farm level but rather on the slaughter level. Accordingly, in terms of input values for the QMRA, Scenario 1 and Scenario 2 do not differ in the prevalence (known slaughter pig prevalence vs. reduced prevalence for 100% implementation rate) but in the value describing fecal leakage at slaughter.

### 2.2 Cost of BSM implementation

The implementation costs were estimated for each BSM individually and included, according to the BSM’s definition, cost parameters, such as labor, equipment, material, and maintenance. The exact implementation definitions of the specific BSMs were derived from the scientific literature used in the evaluation of pathogen-specific BSM effectiveness in the above-mentioned meta-analysis (38). Additionally, national biosecurity guidelines, legal frameworks and expert opinions from the previously established BIOPIGEE expert panel were consulted. The costs of BSMs were assessed under consideration of the specific price indices for the year 2019 to ensure consistency throughout the study. Country-specific cost data were used for both evaluated countries, provided this information was available.

Determined by the different targets of the BSMs, the following cost units were used to estimate the corresponding one-year expenditures: (i) farm, (ii) slaughter pig, and (iii) breeding sow. (i) The cost calculation for farm-level BSMs was conducted based on a country-specific average pig farm, using individual parameters (i.e., farm size, average number of employees, and number of different sections on a farm) based on expert opinion. (ii) The slaughter pig-associated costs were calculated per fattening pig, with the country-relevant information on pig population provided by the national slaughter statistics. (iii) For two BSMs, namely the disinfection of farrowing pens after each farrow, and vaccination, the BSM-related costs were assessed per breeding sow and determined by the country-specific size of the sow population and their reproductive performance (Table 2).

**Table 2:** Country-specific pig industry data used for the assessment of cost associated with the implementation of biosecurity measures.

Cost unit	Country	Number of cost units	References
Farms (*1,000)	AT	21.1	(44)
	UK	10.5	(45)
Slaughter pigs (*1,000)	AT	5002.8	(44)
	UK	10862.1	(46)
Breeding sows (*1,000)	AT	230.2	(44)
	UK	404.0	(46)

The cost calculation for each evaluated BSM x , at the cost-unit level can be simplified and expressed through the following equation (Eq. 2), with BSM-specific cost $C_{u,x}$ , depending on the parameters $P_{u,i,x}$ of the analyzed BSM x , i.e., the corresponding unit u (e.g., farm), specific implementation-associated parameters i (e.g., material, labor), and their costs $c_{u,i,x}$ :


(2) $$C_{u,x}=\sum_{i=1}^{n}P_{u,i,x}\;.\;c_{u,i,x}$$

The total cost for increasing the pre-existing implementation rate $f_{x}$ for each BSM x to countrywide 100% implementation rate is expressed as $C_{T,x}$ and can be obtained by multiplying the unit costs by the total number of respective units $N_{u}$ , identified for the specific BSM, and the pre-existing implementation rate $f_{x}$ , as listed in Table 1 (Eq. 3):


(3) $$C_{T,x}=C_{u,x}\;.\;N_{u}\;.\;\left(\;1-f_{x}\right)$$

Individual calculations for all BSMs evaluated, including parameter values and details can be found in the Supplementary Tables S1, S2.

### 2.3 Identification of disease-associated costs

**Figure 2:**
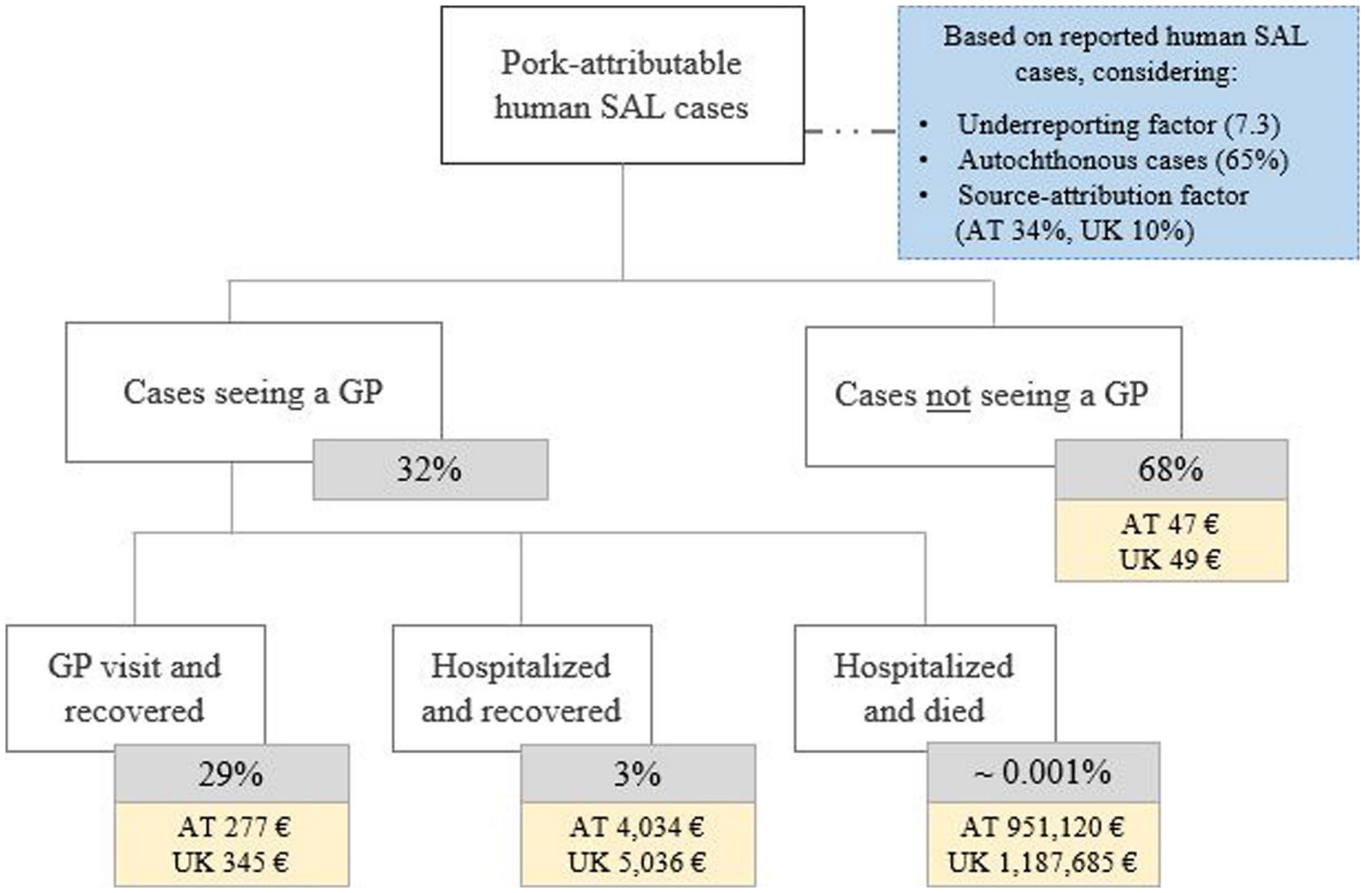
Cost of illness analysis for *Salmonella* (SAL) infection in humans. While SAL-specific factors (blue box) determine the total number of considered human cases, specific severity classes were identified to calculate the average costs per case across these classes. These estimates were based on a previously established methodology comprehensively described elsewhere (9). The average weighted costs per severity level considers both direct and indirect costs combined as described in chapter 2.3. GP, general practitioner; AT, Austria; UK, United Kingdom.

Costs per human salmonellosis cases were evaluated by applying the COI methodology. For this purpose, incidence data were derived from national public health statistics whereas only losses due to primary diseases were considered. The economic burden per infection was expressed as a weighted average across all considered severities. The methodology and the values from an extensive SAL-specific COI analysis (9) were adjusted for the countries evaluated in the economic analysis (Figure 2). The method examined four different severity outcomes: mild cases that recovered without seeing a general practitioner; those that recovered with seeing a general practitioner; hospitalized and recovered cases; and hospitalized and deceased individuals (9). Both direct (e.g., doctor visits, treatment) and indirect costs (e.g., absenteeism from work) were included in the COI analysis. To consider not only incidences related to the consumption of pork meat but to include all infections within the community, both a source-attribution factor (SAF), dependent on the geographic region (AT 0.34; UK 0.10) (47), and an underreporting factor (URF) (both AT and UK: 7.3) (48), were considered in the adjustments of the countrywide reported human salmonellosis cases. Additionally, a factor identifying only those infections which were locally acquired (AT, UK 65%) (7) was applied. Based on these considerations, average overall cost values ranging from €980 (AT) to €1,217 (UK) per human salmonellosis case could be obtained by using the above mentioned parameters derived from literature in the established methodology (9) (Table 3). Analyzing the costs resulting from the illness in pigs, the previously estimated corresponding value (9) was adjusted to the year 2019 and the burden was identified to be €1.77 per pig for both evaluated countries.

**Table 3:** Estimates on the number and costs of pork-attributable human cases used in the cost of illness analysis.

Country	Reported human cases	Estimated human cases^a^	Average cost per human case^b^ (€)	References for reported human cases
AT	1,868	3,023	980	(49)
UK	9,718	4,888	1,217	(7)

The number of human cases is based on statistical reports and the additional parameters listed below. AT, Austria; UK, United Kingdom.

a An underreporting multiplier factor (7.3), a domestic-acquired factor (65%), and a country-specific source-attribution factor (AT 34%; UK 10%) were considered.

b Average cost per human case considering all severity classes and calculated according to the Food Control Consultants Consortium (9) and the country-specific parameters identified in the public health sources described in 2.3 and shown in Figure 2.

### 2.4 Cost-benefit analysis

Subsequently, a CBA was conducted to evaluate the actual economic cost-effectiveness of the BSMs. This method allows a comparison between the cost attributed to the implementation of the specific BSMs and the benefit, representing the avoided costs identified in the COI analysis, including the losses associated with salmonellosis in pigs. For human cases, the benefits were calculated based on the reported national incidence data and the relative reduction in human incidence due to the implementation of the BSM x as derived from the QMRA outputs. For pigs, the relative reduction in prevalence which served as the basis for estimating the benefits is described in detail in chapter 2.1 and Supplementary Information 1.2. The output parameters of the cost-benefit analysis, i.e., the BCR and net benefit were calculated for each BSM and country individually.

The BCR (Eq. 4)


(4) $${BCR}_{x}=\frac{B_{T,x}}{C_{T,x}}$$

weighs the total benefit $B_{T,x}$ against the total cost $C_{T,x}$ of a BSM x . $B_{T,x}$ encompasses the benefits on the public health side incurred due to avoided human cases and the avoided losses in animals associated with a lower prevalence in the pig population.

Therefore, $B_{T,x}$ can be described as follows (Eq. 5):


(5) $$B_{T,x}=B_{H,x}+B_{P,x}\text{,}$$

with Eq. 6 identifying the benefits due to the effect of BSM x within the human population $B_{H,x}$ :


(6) $$B_{H,x}=C_{c}\;.\;H_{C}\;.\;R_{HC,x}\text{,}$$

where $C_{C}$ describes the cost per human case, $H_{C}$ the number of countrywide reported human infections due to the consumption of pork meat acquired within the home country, and $R_{HC,x}$ refers to the relative reduction in human incidence due to the implementation of the BSM x along the PPC derived from the QMRA.

Additionally, (Eq. 7) determines the benefits incurred along the PPC, $B_{P,x}$ as:


(7) $$B_{P,x}=C_{PP}\;.\;P_{PP}\;.\;R_{PP,x}\text{,}$$

where $B_{P,x}$ is described through the parameters $C_{PP}$ , the costs per positive pig $P_{PP}$ , the number of positive pigs in the population, and $R_{PP,x}$ , the relative reduction of positive pigs in the population due to the applied BSM x .

The total cost $C_{T,x}$ depends on the individual BSM x under evaluation and the existing implementation rate as described in (Eq. 3). The net benefit $NB_{x}$ , which provides an absolute measure of benefits is expressed as the difference between $C_{T,x}$ and $B_{T,x}$ (Eq. 8):


(8) $${NB}_{x}=B_{T,x}-C_{T,x}$$

### 2.5 Uncertainty analysis

Uncertainty in the QMRA outputs due to sampling error (50) is included explicitly in the CBA, giving rise to BCR with associated 95% CI. However, all other sources of uncertainty were considered in a separate uncertainty analysis (UA), which is described below. The following aspects were included in the UA: (i) the effectiveness of the BSM (expressed in the 95% CI of the respective RR), (ii) the implementation-cost evaluation of the BSM, and (iii) the disease-cost evaluation in humans and pigs. (i) The CI of the RR was used in the QMRA to assess the uncertainty related to the effectiveness evaluation of an individual BSM, which was conducted for those BSMs initially identified as cost-effective (Table 4). (ii) Relevant parameters within the cost evaluation were assessed by conducting the calculations based on minimum and maximum values given by experts or by decreasing and increasing the given value by 20% (27). Only parameters were selected for which the experts were not in agreement, which were based on estimates or for which there was no reliable evidence in the published literature (Table 4). (iii) Additionally, the uncertainty resulting from cost estimates for human cases and infected pigs was evaluated. Alternative values identified in published literature (Table 4) were used to analyze the impact of changes in the cost per pig case. The originally used value of €1.77 was increased to €13.22 in AT and €13.32 in the UK (52), including revenue losses per animal. Similarly, the costs for human salmonellosis cases were increased from €980 to €1,453 in AT and from €1,217 to €1,464 in the UK (51).

**Table 4:** Parameters considered in the uncertainty analysis (UA) of the evaluated biosecurity measures (BSMs).

		Austria		United Kingdom	
		Original value	Value used in the UA	Original value	Value used in the UA
Cost of disease	Factor				
Cost per human case^a^	Cost (€)	980	1,453	1,217	1,464
Cost per pig case^b^	Cost (€)	1.77	13.22	1.77	13.32
BSM	Parameter^c^				
Organic acid in feed, weaner	RR (median) Amount of feed per weaner^d^ (kg)	0.528 40	min: 0.300 max: 0.931 min: 32 max: 48	0.528 64	min: 0.300 max: 0.931 min: 51.2 max: 76.8
Organic acid in water	RR (median) Amount of water per day (L)	0.250 2	min: 0.088 max: 0.710 min: 1.6 max: 2.4	0.250 2	min: 0.088 max: 0.710 min: 1.6 max: 2.4
Anal plugging	Annual labor cost (€)	28,961	min: 23,169 max: 34,754	28,961	min: 23,169 max: 34,754
Disinfection of farrowing pens	RR (median) Time per disinfection (min) Hourly labor cost (€)	0.465 2 34.7	min: 0.302 max: 0.716 min: 1 max: 3 min: 27.8 max: 41.6	0.465 2 28.5	min: 0.302 max: 0.716 min: 1 max: 3 min: 22.8 max: 34.2

Four BSMs shown to be cost-effective in both Austria and the United Kingdom were analysed. Presented are the original values used in the cost-benefit analysis, and BSM-specific parameters with their minimum (min) and maximum (max) values for the UA. RR, risk ratio.

a Literature-based, alternative value for cost per human case (51).

b Literature-based, alternative value for cost per pig case (52).

c Intervention-specific parameters, which are not based on market values, were selected for the UA.

d Country-specific values are based on expert opinion. The difference can be explained by variation in the weaning-period duration in Austria and the United Kingdom.

## 3 Results

### 3.1 Cost-effectiveness evaluation

The results of the CBA are presented in detail in Table 5. Four of the nine BSMs, namely organic acid in feed (weaner), organic acid in water, anal plugging, and disinfection of farrowing pens were cost-effective for both evaluated countries. In addition, vehicle wheel disinfection resulted in a BCR > 1 for the UK, but was not cost-effective for AT. The BCRs for all other BSMs in both countries were <1, indicating that under the current assumptions, these BSMs are not cost-effective in the reduction of SAL along the PPC.

**Table 5:** Results of the cost-benefit analysis on implementation of biosecurity measures (BSMs) in Austria and the United Kingdom.

Cost-benefit analysis results						
Austria						
BSM	Cost unit^a^	Cost per unit (€)	Total cost (€ million)	Total benefit (€ million)	Benefit-cost ratio median [95% CI]^b^	Net benefit (€ million)
Organic acid in feed, fattener	SP	0.7	2.3	0.5	0.233 [0.172;0.294]	−1.7
Organic acid in feed, weaner	SP	0.1	0.4	1.1	2.648 [2.352;2.945]	0.7
Organic acid in water	SP	0.4	1.9	2.2	1.197 [1.154;1.240]	0.4
Anal plugging	SP	0.4	1.9	2.8	1.418 [1.392;1.445]	0.8
Boot disinfection	Farm	2,842	43.9	0.4	0.010 [0.006;0.013]	−43.5
Disinfection of farrowing pens	BS	9.8	0.9	1.0	1.124 [0.995;1.253]	0.1
Rodent control	Farm	1,304	14.0	0.9	0.063 [0.054;0.071]	−13.2
Vaccination^c^	BS	11.7	18.5	1.9	0.105 [0.100;0.110]	−16.6
Vaccination^c^	SP	3.2				
Vehicle wheel disinfection	Farm	623	13.1	1.6	0.125 [0.118;0.132]	−11.4

United Kingdom						
BSM	Cost unit^a^	Cost per unit (€)	Total cost (€ million)	Total benefit (€ million)	Benefit-cost ratio median [95% CI]^b^	Net benefit (€ million)
Organic acid in feed, fattener	SP	0.8	5.1	1.6	0.318 [0.030;0.332]	−3.5
Organic acid in feed, weaner	SP	0.2	1.4	3.7	2.661 [2.615;2.708]	2.3
Organic acid in water	SP	0.4	4.1	8.3	2.035 [2.028;2.043]	4.2
Anal plugging	SP	0.4	3.9	5.5	1.418 [1.412;1.423]	1.6
Boot disinfection	Farm	2,600	20.1	1.7	0.084 [0.081;0.088]	−18.4
Disinfection of farrowing pens	BS	8.3	1.3	3.4	2.600 [2.546;2.644]	2.1
Rodent control	Farm	1,304	7.0	3.3	0.478 [0.469;0.486]	−3.7
Vaccination^c^	BS	11.7	39.1	7.1	0.182 [0.181;0.183]	−31.9
Vaccination^c^	SP	3.2				
Vehicle wheel disinfection	Farm	567	1.6	2.6	1.631 [1.591;1.671]	1.0

CI, confidence interval.

a The cost units are BSM-specific (SP: slaughter pig; BS: breeding sow; farm).

b Uncertainty interval associated with the sampling error resulting from the QMRA simulations (50).

c For vaccination, the costs per BS and piglet were calculated separately. In a further step, the total cost and benefit were evaluated.

The BSMs which have been shown as cost-effective for both countries were implemented at the animal level (i.e., breeding sow or slaughter pig) and were associated with the lowest total cost among the BSMs evaluated, ranging from €0.4 to €1.9 million for AT and from €1.3 to €4.1 million for the UK. For AT, implementing organic acid in feed (weaner) resulted in the highest BCR (median [95% CI]) 2.648 [2.352; 2.945], followed by anal plugging (BCR: 1.418 [1.392; 1.445]), and organic acid in water (BCR: 1.197 [1.154; 1.240]). For the UK, the most cost-effective BSM was organic acid in feed (weaner) with a BCR of 2.661 [2.615; 2.708], followed by disinfection of farrowing pens (BCR: 2.600 [2.546; 2.644]), and organic acid in water (BCR: 2.035 [2.028; 2.043]). The highest total benefit resulting from the BSM implementation was associated with anal plugging (AT = €2.8 million; UK = €5.5 million), organic acid in water (AT = €2.2 million; UK = €8.3 million), and vaccination (AT = €1.9 million; UK = €7.1 million). The highest net benefit values were reached for anal plugging for AT (€0.8 million) and for organic acid in water for the UK (€4.2 million).

Determined by the high total implementation cost, the BSMs with the lowest BCR were boot disinfection (BCR: AT = 0.010 [0.006; 0.0013]; UK = 0.084 [0.081; 0.088]), vaccination (BCR: AT = 0.105 [0.100; 0.110]; UK = 0.182 [0.181; 0.183]) and rodent control (BCR: AT = 0.063 [0.054; 0.071]; UK = 0.478 [0.469; 0.486]). Additionally, in correspondence to their high total cost, these BSMs had negative net benefit values.

Out of the evaluated BSMs, only anal plugging is to be applied in the post-harvest section during the slaughter process, thus considering only benefits from the reduction in human cases. However, the total net benefit of this BSM for AT (€0.8 million) was even higher than that of organic acid in feed (weaner) (€0.7 million), which was, according to the results, the BSM with the highest BCR.

The BSMs evaluated at the farm level, i.e., rodent control, boot disinfection and vehicle wheel disinfection (for AT) carried high total cost between €7 and €44 million per year and resulted in comparatively low benefit, thereby not suggesting cost-effectiveness. Similarly, for the UK, all farm-level BSMs except vehicle wheel disinfection were shown not to be cost-effective (Table 5).

### 3.2 Uncertainty analysis

While the UA was conducted for all evaluated BSMs, detailed results are only presented for the four BSMs that were cost-effective for both AT and the UK (Table 6). When assessing the identified uncertainty of individual parameters associated with the non-cost-effective BSMs, only an increase of disease-associated cost in pigs generated a BCR > 1 for rodent control in the UK. No further changes leading to cost-effectiveness in the non-cost-effective BSMs could be observed.

**Table 6:** Benefit-cost ratio (BCR) distributions based on the uncertainty analysis (UA) of the cost-effective biosecurity measures (BSMs).

BSM	Austria		United Kingdom	
	Original BCR	UA BCR range^a^	Original BCR	UA BCR range^a^
Organic acid in feed, weaner	2.648	[0.444;4.354]	2.661	[0.369;11.100]
Organic acid in water	1.197	[0.444;1.756]	2.035	[0.758;8.404]
Anal plugging	1.418	[1.225;2.102]	1.418	[1.229;1.706]
Disinfection of farrowing pens	1.124	[0.397;1.649]	2.595	[1.138;10.811]

a The ranges take into account the uncertainty in the effectiveness of the BSMs, uncertainty regarding cost parameters, including alternative values for cost of disease.

The estimated BCR distributions reflect the identified uncertainty in the epidemiological evaluation and economic analysis (Table 6). Considering the uncertainty of the RR-based effectiveness of BSMs by simulating the lowest possible effect within the CI of the RR resulted in a BCR < 1 for all evaluated BSMs, except for the implementation of the disinfection of farrowing pens in the UK. The results showed that lower effectiveness of the cost-effective BSMs using organic acid, as identified in the scientific literature, would lead to BCR values that would no longer indicate cost-effectiveness (organic acid in feed (weaner): AT: BCR = 0.444; UK: BCR = 0.369 and organic acid in water: AT: BCR = 0.444; UK: BCR = 0.758). No cost-effectiveness was identified for the implementation of the disinfection of farrowing pens, once the reduced effectiveness of the BSM was evaluated for AT (BCR = 0.397). However, for the UK the BSM remained cost-effective despite consideration of a lower effectiveness (BCR = 1.138). For the BSM anal plugging, the UA of the RR-based effectiveness could not be conducted, as this BSM was included in the QMRA based on a different approach described in chapter 2.1. Thus, only the variability of COI-related parameters was considered here.

The increased values (Table 4) identified from the literature for the costs associated with SAL infection in pigs (AT €13.22; UK €13.32) contributed to higher benefits and thereby higher BCR values. Compared to AT, the increase in BCR was higher for the UK, i.e., up to 4 times higher than with the original cost per pig of €1.77, whereas for AT, the respective BCR value was only 1.2 times higher. Additionally, the higher costs per human salmonellosis case, including sequelae, led to a 47% increase in the BCR values for AT, while the increase in BCR for the UK was significantly lower at 13%.

Varying the input values of the parameters, e.g., URF or SAF used to estimate the total number of human cases within the COI analysis, or modifying the evaluated cost per case by +/− 20%, resulted in a proportional relative change in the output. The underlying multiplication identifying the benefit due to the pork-attributed avoided human cases resulted in the output showing the same relative change.

Overall, the greatest impact on the BCRs was observed due to variations in COI calculations, when higher costs per human case were considered in AT and higher costs associated with SAL infection in pigs were considered in the UK (Tables 4, 6).

## 4 Discussion

The study presented here aimed to assess the cost-effectiveness of specific BSMs that are shown to be effective in reducing SAL prevalence along the PPC. The results of the analysis indicated the cost-effectiveness of four of these BSMs in AT and five in the UK.

The economic analysis conducted in this study is built on the epidemiological outputs of the QMRA model and reflects the monetary impact resulting from the implementation of the BSMs. Their effectiveness inputted to the model is based on the results of the above-mentioned meta-analysis focusing on biosecurity in pig farms (38). The ORs delivered from this analysis were converted to RRs to meet the technical requirements of the QMRA model (28), although this conversion resulted in a reduction in the total number of BSMs available for the economic evaluation. While ORs and RRs are common risk identifiers in epidemiological or clinical studies (53, 54), some authors used other methods to define reduction values in the simulation models (36, 55, 56). Using the RRs of the BSMs introduced additional uncertainty into the study, which was reflected in the UA. The UA results showed that an inaccurate estimate of the BSM’s effectiveness could reverse its cost-effectiveness. The RRs only consider the direct effect of the interventions but exclude possible implementation shortcomings, which could not be included in the epidemiological evaluation. To reduce uncertainty, more comprehensive information on the effectiveness of the BSMs should be obtained. In addition, the effectiveness of some BSMs has been evaluated at the herd or farm level, i.e., not at the individual pig level. Since the individual farms in which the effectiveness of BSMs was evaluated differ in their structure, management and hygiene, despite national and international regulations the uncertainty associated with these differences cannot be eliminated at this point. However, the resulting BCRs are still informative, since they represent a best-case scenario, based on the understanding that the RR takes its maximum value when evaluated at the individual pig level (28). Hence, BSMs identified as not cost-effective under the current considerations would not be expected to have a BCR > 1, even if RRs at the individual pig level were available (28).

The design and input data requirements of the QMRA model have a major impact on the structure of the presented economic analysis and the certainty of its output. The architecture of the model allows the evaluation of only one specific pathogen at a time (13, 28). However, it is expected that BSMs would benefit a wide range of pathogens (including endemic diseases of pigs), making positive reductions in both pig and human cases of disease. Models encompassing the transmission risk of multiple pathogens would enable the evaluation of an expected higher cost-effectiveness of BSMs that are effective for more than one pathogen. This could promote a holistic approach to such analyses, as for example public health data already indicates a correlation between SAL and other immunomodulatory viruses, with relevance to general human and animal health (57). Further, in our study, only a 100% implementation rate of BSMs was considered in the QMRA model, which has previously been indicated as a constraint in the cost assessment of BSMs (24). Continuous application of BSMs to 100% of farms or animals has a direct impact on the total cost, which could be reduced by their implementation in targeted herds only, e.g., those contributing most to the overall prevalence. However, QMRA does not have the capability to simulate this, because it treats all herds as having the same prevalence. Reducing the countrywide implementation rate in QMRA would lead to a linear scaling of the cost and, similarly, benefits. An option to consider only large farms would allow comparison of complex implementation strategies and support cost-sensitive decisions more effectively. Furthermore, the implementation rate derived from the above-mentioned questionnaire was not country-specific but estimated for both evaluated countries together. Such an approach ignores the regional characteristics of farms and the impact of national legal frameworks for establishing biosecurity strategies. For future epidemiological-economic studies with focus on higher-risk farms, the necessary baseline data should be collected at the country level.

While it is debatable whether the use of organic acid to reduce specific gastrointestinal pathogens can be considered as a BSM (16), the application appears to be cost-effective at certain production stages (24, 58). According to our analysis both BSMs using organic acids, either in feed for weaners or added to water, achieved a BCR > 1 even if applied to 100% of the slaughter pigs. The evaluated costs per pig for adding organic acid into the weaner’s feed (€0.14/pig AT; €0.22/pig UK) are the lowest costs incurred per pig among the analyzed BSMs. While the country-specific length of the weaning period, as well as the required amount of water or feed for this production stage, were considered along with the price of the acid, additional costs due to possible corrosive damage of the feeding or watering pipes could not be included in the analysis. An underestimation of the total cost can therefore not be excluded. Moreover, possible negative effects such as the development of acid tolerance (59) were not considered in the CBA, since the risk for its development is not conclusively supported in the literature (60). Due to data gaps in the assessment of benefits based on performance indicators, potentially improved weight gain due to organic acid intake (61) was not assessed in our study.

According to our results, disinfection of farrowing pens was shown to be a cost-effective BSM when implemented in AT or the UK. However, carrying out disinfection at the entire farm level has previously been identified as not cost-effective (24, 25). In our study, we specifically calculated the costs of disinfecting farrowing pens after every use for each breeding sow (AT €9.8/sow per year; UK €8.27/sow per year). In this cost estimate, the number of all breeding sows in a country and their reproductive performance were taken into account, but the national replacement rates were not considered due to a lack of specific data. Therefore, an overestimation of the total cost is possible.

Anal plugging, the purpose of which is to effectively reduce fecal contamination during the slaughter process (62), was identified as another cost-effective BSM to reduce SAL along the PPC and is the only BSM analyzed to be implemented in the slaughterhouse. In general, BSMs seem to be more cost-effective when applied during the slaughter process (25, 26, 36, 55). Various associated costs, including labor as well as general and specific equipment, were taken into account to determine the total cost per slaughter pig (€0.38/pig AT; €0.35/pig UK). Since anal plugging was not studied under field conditions, data were lacking to consider a potential reduction in the processed carcasses per day and additional costs. Furthermore, the QMRA based estimation of the effect of anal plugging assumed that the plugs seal the anus completely in 100% of the pigs. Thus, the current estimate might overestimate the benefit of this BSM. Future evaluations should include these considerations to reduce the uncertainty of the estimates. If vaccination could be applied in a more targeted manner, such as at nucleus and multiplier farms at the top of a PPC, then this may improve the cost-effectiveness.

Although the effectiveness of the other BSMs evaluated to reduce SAL was demonstrated in the meta-analysis (38), their respective BCRs were <1 for both countries. Even though the effectiveness of vaccination of pigs to reduce the exposure to humans was proven, i.e., a case reduction of 63% (41), the calculated BCR suggests that implementation in 100% of animals is not cost-effective. This BSM resulted in the highest total cost for a one-year implementation when applied to all sows and all piglets within a 1 year cycle in the UK (€39.1 million).

For the important factors related to the benefits, total benefit is most influenced by the costs associated with human salmonellosis, i.e., AT (~97%) and UK (~57%). Although the results for some parameter variations within the COI analysis are not presented in the UA results section, the importance of these factors should still be discussed due to the direct relative change in the economic output. The URF (7.3) used for human SAL cases is not country-specific (48). European studies have used different multiplication factors ranging from 4.7 to 57.5 to estimate the total number of salmonellosis cases in the community (36, 52, 63–66). Several aspects may have an influence, such as the national health system, the accessibility and availability of resources, and the individual’s willingness to seek medical attention (51, 67). Disease pyramids including critical points where positive cases could be missed (51) should be evaluated for each country individually based on national information from various sources. The SAF used in this study for salmonellosis (47) has been calculated for specific geographic regions only, i.e., for Western Europe (34.1%) and Northern Europe (10.6%), which introduces uncertainty in our country-specific analysis. Other CBA studies used higher values for the UK (24) and lower values for AT (48). Different consumer habits and national product availability, such as for cured pork sausages, influence the national SAF as well. While individual studies on national SAF values are available for some countries (68–70), they were not identifiable for the countries evaluated within this study. Country-specific assessments rely on disease-outbreak data and comparison of SAL serovars in the potential source and human cases when a microbial subtyping is applied (47). Future studies should attempt to establish country-specific SAFs, taking local consumption patterns into account. Additional factors, notably antibiotic resistance, which is a rising concern in SAL serovars common in pigs (71), economic changes on the European pork market (72), and the consideration of multiple affected livestock species as sources (51), should be included in future animal health economics research. Moreover, increased consumer risk awareness and improved hygiene in households during meat processing could contribute to the reduction in the number of human infections.

Any major disease outbreak in meat-producing livestock affects the meat market and may have consequences for trade. The EU is a single market without borders and therefore special considerations should be included when assessing the impact of foodborne diseases on trade in Europe (72). Surveillance frameworks are essential for such a market system, however, there are no standardized and regulated surveillance systems for SAL along the PPC in place in both evaluated countries, and there is a lack of information on the associated expenditure. Therefore, the effectiveness of surveillance and the associated costs and benefits could not be considered in our study. Similarly, no data is available on the number and magnitude of pork-attributed outbreaks across Europe, nor domestic product recalls, which would be necessary to investigate possible corresponding market changes, e.g., in supply, demand and prices. Despite the existence of the Common market, there is a considerable variation in pork market characteristics between Member States. Moreover, pork prices are characterized by seasonality, randomly occurring fluctuations and various short- and long-term trends (73). Therefore, respective considerations of market effects resulting from SAL occurrence on pig farms are subject to high uncertainty.

One of the general challenges when implementing biosecurity systems is the undeniable discrepancy between the stakeholder carrying the costs versus those benefiting from the outcome (36). Farmers make relevant decisions based on potential value added and the ease and costs of the implementation (74). The possibility of considering multiple pathogens, mentioned above, could help move these discussions forward. While penalties for high SAL prevalence have been introduced in some European countries (75, 76), visible financial benefits within their production system might still be the most convincing factor for farmers. Other diseases in pigs, the incidence of which can be reduced by the implementation of BSMs, such as post-weaning multi-systemic wasting syndrome and porcine circovirus 2, can result in high losses for farmers (77), and therefore the potential additive reduction-effect of BSMs needs to be evaluated.

Nevertheless, the motivation to prevent Salmonellosis in humans through biosecurity should not be determined solely by the cost-effectiveness of the BSMs based on the COI approach, which is limited to measuring the losses in monetary terms. It is arguable, whether personal disease burdens such as suffering, pain, and loss in productivity can be captured in monetary terms, and therefore methods estimating the burdens in non-monetary ways should be considered too. Such methods are, e.g., Disability-Adjusted Life Year (DALY) or Quality-Adjusted Life Year (QALY), which however, do not include aspects such as the costs associated with the use of health care services (9). When deciding on the implementation of BSMs, stakeholders should therefore consider different approaches to achieve the desired outcomes.

Overall, the current spread of the African swine fever virus in Europe (78) highlights the need for increased biosecurity along the PPC. The concerns on the associated serious economic losses could be an incentive for national authorities and farmers to invest collaboratively in improving biosecurity. In return, targets to reduce SAL in the pig population could be achieved at the same time. Hereby, our results contribute to the discussion on increasing biosecurity along the PPC in order to reduce foodborne disease outbreaks and prevent the spread of infectious animal diseases in general.

## Data availability statement

The original contributions presented in the study are included in the article/Supplementary material, further inquiries can be directed to the corresponding author.

## Author contributions

CB: Conceptualization, Data curation, Formal analysis, Investigation, Methodology, Validation, Writing – original draft, Writing – review & editing. AK: Conceptualization, Funding acquisition, Methodology, Project administration, Resources, Supervision, Validation, Writing – review & editing. NW: Data curation, Formal analysis, Investigation, Methodology, Software, Validation, Visualization, Writing – review & editing. GCC: Data curation, Formal analysis, Investigation, Methodology, Software, Validation, Writing – review & editing. TM: Conceptualization, Formal analysis, Methodology, Supervision, Validation, Visualization, Writing – original draft, Writing – review & editing.

## Glossary

AT: Austria
BCR: Benefit-cost ratio
BSMs: Biosecurity measures
CBA: Cost-benefit analysis
CI: Confidence interval
COI: Cost of illness
EU: European Union
ORs: Odds ratios
PPC: Pork production chain
QMRA: Quantitative microbiological risk assessment
RRs: Risk ratios
SAF: Source-attribution factor
SAL: Salmonella
UA: Uncertainty analysis
UK: United Kingdom
URF: Underreporting factor
