# Supplementary material for: Identification of cost-effective biosecurity measures to reduce Salmonella along the pork production chain
Source: Front Vet Sci. 2024 Mar 18;11:1380029. doi: 10.3389/fvets.2024.1380029 (PMC10983795; doi:10.3389/fvets.2024.1380029)
Supplement: Supplementary file 1 [file Table_1.DOCX]

Supplementary Material

# Supplementary Information

## Estimating risk ratios

For all evaluated biosecurity measures (BSMs) except vaccination and anal plugging, the risk ratios (RRs) were calculated using data from a systematic review and meta-analysis presented elsewhere (1). For vaccination, the RR was taken from Peeters et al. 2020 (2). The effect of anal plugging was considered at the level of the parameters directly in the quantitative microbiological risk assessment (QMRA) model describing the slaughter process. The corresponding model parameter describing fecal leakage through the anus was set to zero.

First, the methodology of estimating RRs using data from the systematic review and meta-analysis is described. Based on the results, the BSMs proven effective in the reduction of *Salmonella* along the pork production chain (i.e., upper limit of identified odds ratio’s 95% confidence interval < 1), were selected from Waller et al. 2023 (1). The RRs were calculated based on the 2x2 contingency table (Table S1), employing the software R version 4.0.3 (3) and the R package metafor version 3.0.2 (4). The function ‘escalc()’ from the metaphor package was used to evaluate Eq.(1):

| $RR=\frac{\frac{a}{a+b}}{\frac{c}{c+d}}$ | (1) |
| --- | --- |
|  |  |

The variables a, b, c, and d correspond to the values of the same designation in the following 2x2 contingency table used to calculate RRs for seven related BSMs. The a, b, c and d are numbers of pigs for the corresponding combination of *Salmonella* occurrence and implementation of a BSM.

Table S1: The basic structure of the 2x2 table used to calculate RRs for seven related BSMs. The a, b, c and d are numbers of pigs for the corresponding combination of *Salmonella* occurrence and implementation of BSM.

|  |  | ***Salmonella*** | |
| --- | --- | --- | --- |
|  |  | YES | NO |
| **BSM** | YES | a | b |
|  | NO | c | d |

## Equation to derive pig prevalence assuming 100% implementation of a BSM

The relative risk for a BSM $x$ (${RR}_{x}$) is defined as (Eq.(2))

| ${RR}_{x}=\frac{p_{x}}{p_{n}}$ | (2) |
| --- | --- |

where $p_{x}$ refers to the prevalence on farms which are implementing the BSM $x$. Since$p_{n}$, the prevalence on farms which are not implementing that same BSM, is unknown, this is substituted by expressing $p_{n}$ in terms of the national baseline prevalence $p$ as follows (Eq.(3))

| $p={p_{x}f}_{x}+(1- f_{x})p_{n}$ | (3) |
| --- | --- |

with $f_{x}$ representing the proportion of farms currently implementing a BSM $x$. Using Eq.(2) in Eq.(3), $p_{n}$ can be eliminated allowing $p_{x}$ to be expressed as (Eq.(4))

| $p_{x}= \frac{p}{f_{x}+ \frac{1-f_{x}}{{RR}_{x}}}$ | (4) |
| --- | --- |

To evaluate $p_{x}$ based on ${RR}_{x}$the observational unit of the risk factor should be “pig”. While this was considered in the literature review to identify effective BSMs(1), the observational units “farm” or “herd” were used in this study too. Although it is not a substitute for the correct prevalence unit, ${RR}_{x}$ can still be considered in the knowledge that any observational unit other than pig would correspond to a smaller RR. Therefore, the results are still relevant, representing the best-case scenario for the BCR of a particular BSM.

Based on the results of (Eq.(4)) we estimated how much the national baseline prevalence would change assuming a 100% implementation rate for BSM $x$. At such a 100% implementation rate the national baseline prevalence would have essentially dropped to a value of $p_{x}$, and so the expected prevalence reduction would therefore correspond to the difference between $p$ and $p_{x}$. Hence the relative reduction in pig prevalence $R_{pp,x}$under the assumption of a 100% implementation rate was calculated according to Eq.(5)

| $R_{pp,x}= \frac{\left( p-p_{x} \right)}{p}\cdot100\%$ | (5) |
| --- | --- |

These values are reported in Table 1 in the main article.

## 2 Supplementary Tables

## Supplementary Table S1. Equations used for estimating the costs of implementing evaluated biosecurity measures (BSMs)

| **BSM** | **Cost equation** | **Cost unit** | **Parameter legend** |
| --- | --- | --- | --- |
| Anal plugging (ap) | $C_{ap}=N_{sp} \cdot(c_{pe}+c_{lap})$ | SP^1^ | N_sp_ … number of slaughtered pigs  c_pe_ … equipment cost for the plug and pusher per slaughtered pig  c_lap_ … labor cost per slaughtered pig |
|  |  |  |  |
|  |  |  |  |
| Boot disinfection (bd) | $C_{bd}=N_{d}\cdot F_{c}\cdot{(c}_{dd}+c_{lbd})+c_{dt}\cdot$ $N_{d}$ | Farm | N_d_ … number of disinfection trays on the farm  F_c_ … factor “change”: 156 (3x/week)  c_dd_ … cost per disinfection dip  c_dt ­_… cost per disinfection tray  c_lbd_ … labor cost |
|  |  |  |  |
|  |  |  |  |
|  |  |  |  |
| Disinfection of farrowing pens (d) | $C_{d}= c_{dl}\cdot\left( L_{af}+L_{aw} \right)+c_{ed}+c_{ld}$ | BS^2^ | c_dl_ … cost per litre disinfectant used  L_af_, L_aw_ … amount of disinfectant for floor and walls needed  c_ed_ … cost for equipment  c_ld_ … labor cost |
|  |  |  |  |
|  |  |  |  |
|  |  |  |  |
| Organic acid in feed, fattener (oaf,f) | $C_{oaf,f}=N_{f}\cdot{OAF}_{f}\cdot c_{oa/u}$ | SP | N_f_ … number of slaughter pigs/year  OAF_f_ … amount of organic acid needed  c_oa/u_ … cost per unit |
|  |  |  |  |
|  |  |  |  |
| Organic acid in feed, weaner (oaf,w) | $C_{oaf,w}=N_{w}\cdot{OAF}_{w}\cdot c_{oa/u}$ | SP | N_w_ … number of slaughter pigs/year  OAF_w_ … amount of organic acid needed  c_oa/u_ … cost per unit |
|  |  |  |  |
|  |  |  |  |
| Organic acid in water (oaw,w) | $C_{oaw,w}=N_{w}\cdot{OAW}_{w}{\cdot c}_{oa/u}$ | SP | N_w_ … number of slaughter pigs/year  OAW_w_ … amount of organic acid needed  c_oa/u_ … cost per unit |
|  |  |  |  |
|  |  |  |  |
| Rodent control (rc) | n/a | Farm | n/a |
| Vaccination piglet (vp) | $C_{vp}= N_{p}\cdot c_{vd}\cdot2$ | SP | N_p_ … number of slaughter pigs/year  c_vd_ … cost per dose |
|  |  |  |  |
| Vaccination sow (vs) | $C_{vs}=N_{s}{\cdot c}_{v}\cdot L_{y}+{(N}_{s}\cdot c_{v})$ | BS | N_s_ … number of breeding sows  L_y_ … average number of litters per year  c_v_ … cost per dose |
|  |  |  |  |
|  |  |  |  |
| Vehicle wheel disinfection (vwd) | $C_{vwd}= N_{vv}\cdot c_{vwd}$ | Farm | N_vv_ … number of visiting vehicles per year  c_vwd_ … cost per vehicle to be disinfected (equipment, labor, time) |

^1^ SP, Slaughter pig

^2^ BS, Breeding sow

**Supplementary Table S2. Data used to calculate the costs of implementing biosecurity measure (BSM) in Austria (AT) and the United Kingdom (UK)**

| **BSM** | **Parameter^1^** | **Unit** | **AT** | **UK** | | **References** |
| --- | --- | --- | --- | --- | --- | --- |
| Anal plugging |  |  |  |  | |  |
|  | Cost per plug | € | 0.08 | 0.08 | | (5) |
|  | Cost per pusher | € | 125 | 125 | | (5) |
|  | Cost per worker | € | 28,961 | 28,961 | | (6) |
|  | Pusher per slaughter pig | € | 0.001 | 0.001 | |  |
|  | Labor cost per slaughter pig | € | 0.3 | 0.3 | |  |
|  | Cost per slaughter pig | € | 0.4 | 0.4 | |  |
| Boot disinfection |  |  |  |  | |  |
|  | Number of dips, average | n | 5 | 5 | | Expert opinion, BIOPIGEE |
|  | Number of dips, small farms^2^ | n | 3 | 3 | | Expert opinion, BIOPIGEE |
|  | Number of dips, large farms^2^ | n | 15 | 15 | | Expert opinion, BIOPIGEE |
|  | Changes per year | n | 156 | 156 | | Expert opinion, BIOPIGEE |
|  | Cost per dip | € | 1.9 | 1.9 | | (7) |
|  | Cost per tray | € | 9.2 | 9.2 | | (7) |
|  | Labor time | min | 3 | 3 | | Expert opinion, BIOPIGEE |
|  | Labor cost | € | 1.7 | 1.4 | | (8) |
|  | Cost per farm, small farms | € | 1,705 | 1,560 | |  |
|  | Cost per farm, large farms | € | 8,527 | 7,802 | |  |
|  | Average cost per farm | € | 2,842 | 2,601 | |  |
| Disinfection of farrowing pens |  |  |  |  | |  |
|  | Size of farrowing pen | m^2^ | 5.5 | 6.5 | | AT: (9); UK: Expert opinion |
|  | Disinfectant cost per litre | € | 19.3 | 19.3 | | (7) |
|  | Equipment cost | € | 223 | 223 | | (10) |
|  | Equipment cost per sow | € | 4.5 | 3.0 | | (10) |
|  | Time per sow/year | min | 4.6 | 4.6 | | Expert opinion, BIOPIGEE |
|  | Labor cost per sow | € | 2.7 | 2.2 |  | |
|  | Cost per sow | € | 9.8 | 8.3 |  | |
| Organic acid in feed, fattener |  |  |  |  |  | |
|  | Amount of acid needed | kg | 0.3 | 0.3 | Expert opinion, BIOPIGEE | |
|  | Cost per kg acid | € | 2.3 | 2.3 | (11) | |
|  | Cost per slaughter pig | € | 0.7 | 0.8 |  | |
| Organic acid in feed, weaner |  |  |  |  |  | |
|  | Amount of acid needed | kg | 0.1 | 0.1 | Expert opinion, BIOPIGEE | |
|  | Cost per kg acid | € | 2.3 | 2.3 | (11) | |
|  | Cost per slaughter pig | € | 0.1 | 0.2 |  | |
| Organic acid in water |  |  |  |  |  | |
|  | Amount of acid needed | kg | 0.1 | 0.1 | Expert opinion, BIOPIGEE | |
|  | Cost per kg acid | € | 3.1 | 3.1 | (7) | |
|  | Cost per weaner | € | 0.4 | 0.4 |  | |
| Rodent Control |  |  |  |  |  | |
|  | Professional Company | € | 1,304 | 1,304 | (12) | |
| Vaccination |  |  |  |  |  | |
|  | Cost per dose/sow | € | 3.6 | 3.6 | (13) | |
|  | Cost per dose/piglet | € | 1.6 | 1.6 | (13) | |
|  | Dosages sow/year | n | 3.3 | 3.3 | Expert opinion, BIOPIGEE | |
|  | Dosages piglet/year | n | 2 | 2 | (13) | |
|  | Cost per sow - 1^st^ year | € | 11.7 | 11.7 |  | |
|  | Cost per piglet | € | 3.2 | 3.2 |  | |
| Vehicle wheel disinfection |  |  |  |  |  | |
|  | Number of visiting vehicles/month (4)^2^ | n | 48 | 48 | Expert opinion, BIOPIGEE | |
|  | Number of visiting vehicles/month (2)^2^ | n | 24 | 24 | Expert opinion, BIOPIGEE | |
|  | Disinfectant cost per vehicle | € | 1.9 | 1.9 | (14) | |
|  | Time per vehicle | min | 15 | 15 | Expert opinion, BIOPIGEE | |
|  | Labor cost | € | 8.7 | 7.1 | (8) | |
|  | Equipment cost | € | 154 | 154 | (14) | |
|  | PPE clothing cost | € | 90 | 90 | (14) | |
|  | Cost per farm (4 visiting vehicles/month)^2^ | € | 749 | 675 |  | |
|  | Cost per farm (2 visiting vehicles/month)^2^ | € | 497 | 460 |  | |
|  | Average cost per farm | € | 623 | 567 |  | |

^1^ Note that parameters listed here represent detailed data, some of which were summarized in the equations in Table S1.

^2^ Values used in the uncertainty analysis.

Calculated values

**References**

1. Waller E, Huber N, Meester M, Sassu EL, Correia-Carreira G, Krumova-Valcheva G. et al. Biosecurity measures reducing Salmonella spp., hepatitis E and pathogenic Escherichia coli prevalence in pig farms: A systematic review and meta-analysis: (Paper under review at Porcine Health Management) 2023.

2. Peeters L, Dewulf J, Boyen F, Brossé C, Vandersmissen T, Rasschaert G et al. Effects of attenuated vaccine protocols against Salmonella Typhimurium on Salmonella serology in subclinically infected pig herds. Vet J 2019; 249:67–72.

3. R Core Team. (2021). R: A Language and Environment for Statistical Computing: R Foundation for Statistical Computing. Retrieved from <https://www.R-project.org/>.

4. Viechtbauer, W. (2010): Conducting meta-analyses in R with metafor package. In: Journal of Statistical Software 36, S. 1–48. DOI: 10.18637/jss.v036.i03.

5. Anonym. Adept Contamination Control Systems; 2022. Available from: URL: <https://www.adeptclip.com/>.

6. Anonym. Talent; 2022. Available from: URL: <https://uk.talent.com/>.

7. Anonym. Desintec; 2022. Available from: URL: <https://www.desintec.de/>.

8. Eurostat. Labour cost levels by NACE Rev. 2 activity 2019; 2022. Available from: URL: <https://ec.europa.eu/eurostat/databrowser/view/LC_LCI_LEV$DEFAULTVIEW/default/table>.

9. Austrian Animal Welfare Legislation; 2022. Available from: URL: <https://www.ris.bka.gv.at/GeltendeFassung.wxe?Abfrage=Bundesnormen&Gesetzesnummer=20003541>.

10. Anonym. OBI Baumarkt; 2022. Available from: URL: <https://www.obi.at/>.

11. Anonym. Garant-Tiernahrung Gesellschaft m.b.H.; 2022. Available from: URL: <https://www.garant.co.at/>.

12. Hecker OC, Boelhauve M, Mergenthaler M. Start-up financing of professional pest control in pig farming in North Rhine-Westphalia in Germany. Porcine Health Manag 2018; 4:22.

13. Anonym. Richter Pharma; 2022. Available from: URL: <https://www.richter-pharma.com/>.

14. Anonym. Desintec: Virkon ® S; 2022. Available from: URL: <https://www.desintec.de/de/desintec_de/produkte/stallhygiene/stallhygiene_86.html>.
